# Value evaluation of cultural tourism tourists’ psychological expectation based on machine learning data mining

**DOI:** 10.3389/fpsyg.2022.943071

**Published:** 2022-08-08

**Authors:** Chih-Hung Pai, Sai Xu, Jianren Jin, Yunfeng Shang

**Affiliations:** ^1^School of Hospitality Management, Zhejiang Yuexiu University, Shaoxing, China; ^2^Center for Eastern Asian Culture Research, Zhejiang Yuexiu University, Shaoxing, China

**Keywords:** psychological expectation, value evaluation, machine learning, data mining, cultural tourism tourists

## Abstract

The era of smart tourism has arrived. In the context of big data information, based on the thinking of the entire tourism activity, it is worth thinking about the role of tourism information in tourism activities. This paper proposes a method for evaluating the psychological expectations of tourist destinations by applying the quality function configuration. According to the needs of tourists, the relevant product characteristics of the tourist destination are selected, an evaluation quality house is established, and various relationships within the quality house are weighed, and established a mathematical model for the evaluation of tourists’ psychological expectations in tourist destinations. Bringing the methods of machine learning (ML) and data mining (DM) into the research of tourists’ psychological expectation value evaluation, ML is one of the main methods to solve the problem of DM. ML is the process of using the system itself to improve itself, therefore, ML is widely used in data mining. The research combines psychology and tourism research, through empirical research, to establish a structural equation model. It analyzes the influence of tourism information on tourists’ behavioral decisions, increases the media’s variable expectations of tourism, and uses tourist satisfaction and behavior as dependent variables. The results showed that the effect of tourism information on tourists is significantly greater than the expected effect (*p* = 0.510, P is significant at 0.001 level) than the effect of tourist satisfaction (*p* = 0.290, P is significant at 0.05 level). Therefore, in order to create good expectations for tourists, the general image of a tourist destination must match the actual local conditions. Using the support vector machine algorithm with the introduction of optimization mechanism to train the feature set of the user data, and then predict the links in Sina Weibo, and obtain higher prediction accuracy and prediction speed. The psychological expectation evaluation model of tourists in tourist destinations can effectively calculate the perceived value of psychological expectation evaluation of tourists in tourist destinations, and help tourists choose reasonable and satisfactory travel plans.

## Introduction

Evaluating the psychological expected value of tourists from the tourist destination is the subjective expectation of tourists on the tourism value of the tourist destination, not the objective value. In a tourist destination, improving tourist satisfaction is to improve the level of product characteristics of the tourist destination. Therefore, choosing tourist destinations according to tourist needs plays an important role in improving tourist satisfaction. The article believes that it is appropriate to choose a reasonable tourist destination according to the needs of tourists (Suanpang et al., 2021b). Cultural tourist attractions are a kind of tourist attractions and an important part of the tourism industry system. There are many ways to achieve tourism experience. Tourism experience is shaped by behaviors such as aesthetics, communication, and consumption. The problem that tourists are difficult to obtain high-level tourism experience deserves attention.

This paper evaluates the psychological expected value of cultural tourism tourists based on ML DM. Machine learning is a way for a system to use accumulated data to improve itself and automatically improve its performance (Suanpang et al., 2021a). A large amount of data has been accumulated in telecommunications, finance, retail, scientific research and other industries and fields, and there are problems and needs of DM. This time, I tried to analyze the open-air mobile terminals in the GSM network, and discussed two typical DM problems of link location and prediction in social networks. On the one hand, the purpose of studying statistical methods of DM is to develop and improve the theoretical basis of DM, and to lay a solid foundation for the development of better and richer DM. Process data guides its decisions and controls it. This paper believes that choosing a reasonable tourist destination according to the needs of tourists plays an important role in improving the quality of tourism. Therefore, it is of great theoretical and practical significance to study the psychological expected value of tourist destinations. However, there are few literatures on the psychological expected value of tourists.

High tourism quality plays an important role, but there is currently little literature on the psychological expected value of tourists. The evaluation of tourists’ psychological expected value of tourist destinations is a complex multi-index decision-making problem. From the perspective of tourists, this paper establishes a mathematical model according to the known product characteristics of different tourist destinations, combined with the personalized needs of tourists, and evaluates the psychological expected value of tourist destinations. It chooses a reasonable travel plan to achieve the highest travel satisfaction and achieve the best allocation of resources. Based on the background of the era of experience, this paper makes a new interpretation of tourism experience from the perspective of psychology, and analyzes the influence of tourism behavior, landscape behavior and environment. In addition to the characteristics and current situation of tourist experience in scenic spots, this paper also puts forward the advantages and disadvantages of improving the tourist experience of cultural scenic spots and promoting the sustainable development of multicultural scenic spots. It analyzes tourists’ perceptions to influence emotion-oriented data, hoping to really explain some tourism events.

## Related work

Experts at home and abroad also have many research results in the evaluation of the psychological expectation value of cultural tourism tourists. Nilashi M proposed a new recommendation method based on multi-criteria CF to improve the prediction accuracy of recommendation systems in the tourism field through clustering, dimensionality reduction and prediction methods (Nilashi et al., 2017). The results of ASH T support the positive moderating effect of resource slack on the relationship between EP and FP individual dimensions in the tourism industry, thus supporting the resource slack hypothesis (Tan et al., 2017). The results of the V Favre-Bont’s study showed that participants in tourist destinations respond to changes in institutional factors by adopting an institutional logic of cooperative competition, which includes five key processes: exploitation, exploration, bridging, sharing, and boundary crossing (Favre-Bonté and Tran, 2018). However, the above research results are not deep enough to analyze the problems contained in the deep level, so the article uses the ML DM method for research.

The purpose of Fong V in ML and DM is to outline methods for predicting solar radiation using ML methods, showing that other methods are starting to be used in this prediction setting (Fong et al., 2018). Altin M believed that computer assistance in synthetic design has been around for more than 40 years, and although it initially appears feasible, the reaction steps often fail when tried in the laboratory (Altin et al., 2018). Chaurasia V believed that the history of DM as a method field can be traced back to exploratory data analysis, and has established methods to determine validity and generality (Chaurasia and Pal, 2017). Varley J B presented the third major version of the KEEL software. KEEL is an open-source Java framework that provides many modules to perform various DM tasks (Varley et al., 2017). Since the research on fir trees has not been verified in practice, it has not been recognized by the public.

## Principles of machine learning algorithms and network positioning of data mining

**FIGURE 1:**
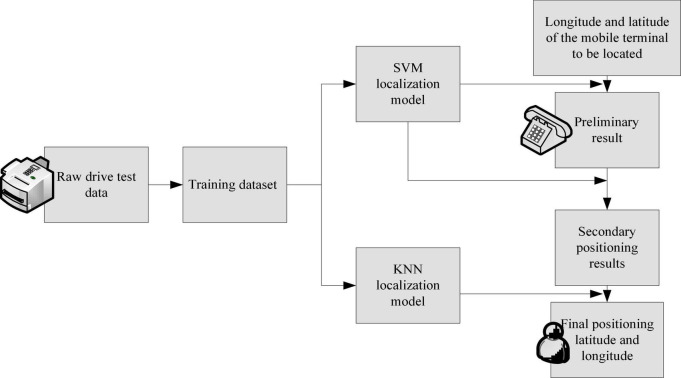
Flow chart of three-stage localization based on SVM and k-nearest neighbor method.

Machine learning is abbreviated as ML, or machine learning. Data mining is abbreviated as DM, that is, data mining. Now use the ML method of protective gear mining to improve the evaluation of cultural tourism tourists’ psychological expected value. Machine learning is an important way to solve DM problems. Its basic idea is to use a large amount of training data to solve the distribution or correction of the decision function to solve the problem, and then make a decision for anonymous samples. This paper mainly summarizes the principle of ML algorithm used in this paper, which is used to solve two specific problems of DM and link prediction of external mobile terminals located in the GSM network. Based on the problem of finding mobile terminals outside the GSM network, a three-stage localization method based on SVM and k-nearest neighbor method was proposed, and compared with the traditional localization method based on SVM and k-nearest neighbor method (Triguero et al., 2017). The flow of the method is shown in Figure 1. This method combines the advantages of the traditional SVM-based positioning method and the k-nearest neighbor positioning method, and makes some improvements to the SVM-based positioning method and the k-nearest neighbor positioning method. It introduces preliminary positioning based on the location of the mobile terminal’s main serving cell, thereby greatly improving positioning accuracy and time consumption. The specific implementation of each step will be introduced below, and the simulation results will be described and analyzed (Buczak and Guven, 2017).

### Modeling of mobile terminal positioning problem based on machine learning

**FIGURE 2:**
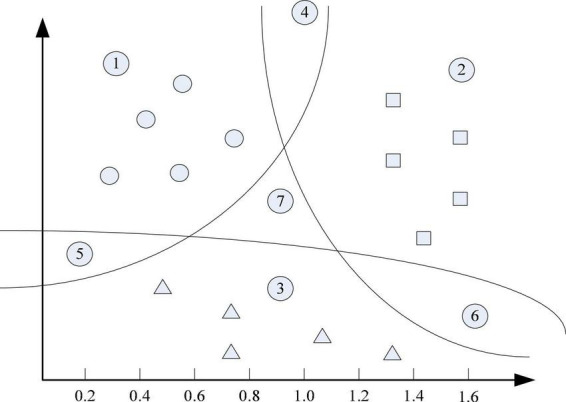
Example of a multi-classification problem.

The deployment method based on vector machine support is to rasterize the deployment area and arrange small grid areas, then it collects a large number of mobile terminal reception level measurement reports on the deployment area, and finally the mobile terminal sends the measurement report. The latitude and longitude levels constitute the training data set. If the mobile terminal needs to be positioned, the similarity level between the horizontal measurement report received from the mobile terminal and the divided grid horizontal measurement report received from the training data set is calculated (Chen and Asch, 2017; Mullainathan and Spiess, 2017). Determine the grid where the mobile terminal is located, transform the deployment problem into a multi-class distribution problem, and introduce ML to solve this distribution problem (Wang et al., 2017). The figure shows an example of solving a multivariate problem using the one-class pair residual method. There are three types of sample points in the Cartesian coordinate system of Figure 2, and the three curves represent the three distribution curves obtained by binary support (Voyant et al., 2017). In the classification of the vector machine, it only needs to change the feature vector from the sample point to the corresponding function on the three curves, and then make a decision based on the value of the function. At this point, we can assume that more sample points come from the corresponding division function curve, and the function value is larger, so they are more likely to belong to the positive class corresponding to the division curve, and the three segmentation curves located at the sample points of area ⑦ equal to the partitioning. All functions require negative values. However, it can also be assumed that the closer the sample points are to the corresponding segmentation function curve, there are two problems with the one-to-remainder method. Even if they are not equal but the difference is not large, the confidence of the judgment result will be greatly reduced. In this case, a threshold of the difference between the division function values should be set, which can alleviate the classification error that may be caused when multiple division functions are all positive values (Zhou et al., 2017). The sample point category cannot be judged. Even if the difference is not equal but the difference is not large, the credibility of the judgment result will be greatly reduced. In this case, a threshold for the difference between the division function values should be set. When there are multiple partitioning functions with positive values, only the difference between them is greater than this threshold, and the classification decision is accepted; otherwise, the one-to-remainder method is considered to be inseparable to the sample points. In this way, the classification error that may be caused when multiple partition functions are positive values can be alleviated to a certain extent (Poret et al., 2018).

### Positioning of samples to be classified

**FIGURE 3:**
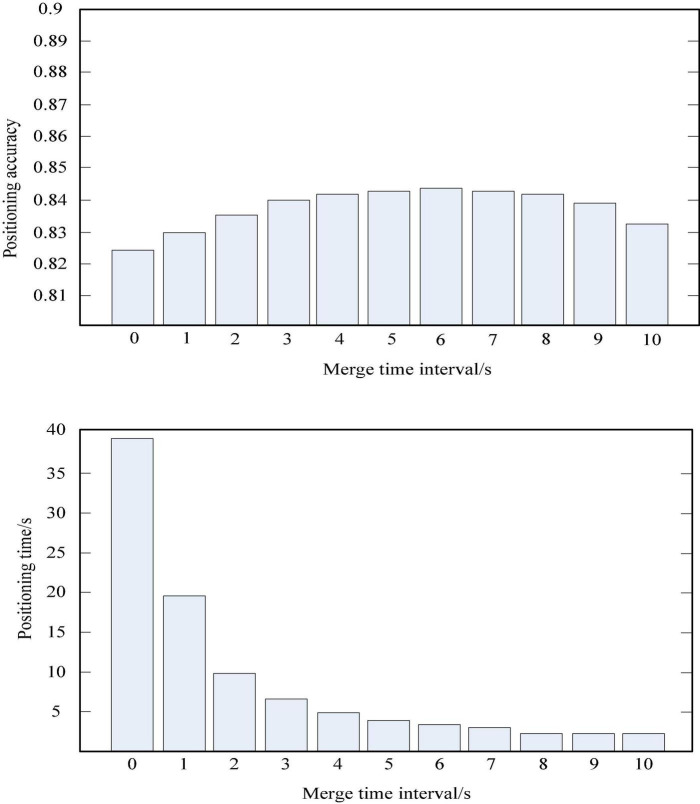
The relationship between the time interval of data merging to be positioned and the positioning accuracy.

First, if the deployment area is determined according to the nearest neighbor k-method in the model, the deployment step based on the nearest neighbor k-method should select the collected training data located in the chosen square area (Liu et al., 2017). The training results obtained during training, based on the steps of the nearest neighbor k-method, since the aggregated training data are computed in the combined region in the order of longitude or latitude priority. Longitude and latitude are from small to large, so it can be a number in the order of latitude and longitude from small to large. The latitude and longitude range of the positioning area can be easily calculated by using the k-nearest neighbor method to locate the latitude, longitude and side length of the center point of the area. Then it will calculate some similarity feature or distance measure with the merged data to be positioned and the selected merged training data during the secondary localization of the backbone SVM. Finally, it selects the k merged training data that are most similar or closest to the data to be located and outputs the average value of the latitude and longitude corresponding to the k merged training data, which is the final positioning result of the three positioning based on the k-nearest neighbor method (Weinan, 2017). The k value of the k-nearest neighbor method should be selected according to the actual simulation results. Generally, there will be an optimal k value to maximize the positioning accuracy (Jain and Kar, 2017). This simulation locates the to-be-located data set containing 1,521,032 drive test data. And it sets the k value of the three positioning based on the k-nearest neighbor method to 30, the side length of the positioning area is set to 300 m, and the side length of the merged area of the training data is set to 10 m to determine the time for merging the data to be located. The positioning performance was simulated when the interval was set to 2 s to 10 s. The simulation results are shown in Table 1 and Figure 3. The percentage of positioning accuracy in the table is the percentage of the specified positioning error within 100 m (Drton and Plummer, 2017). As can be seen from the table, as the time interval for combining the data to be positioned increases, the positioning accuracy will gradually increase, and then slowly decrease after reaching a peak value. The localization speed increases with the increase of the merging time interval (Lamperti et al., 2018).

**TABLE 1:** The results of the combined simulation of the data to be located.

Merge time interval	No	1 s	2 s	3 s	4 s	5 s
Positioning accuracy	82.36%	82.95%	83.5%	83.97%	84.18%	84.27%
Positioning time	39.40 s	19.64 s	9.82 s	6.57 s	4.92 s	3.95 s
Merge time interval	6 s	7 s	8 s	9 s	10 s	
Positioning accuracy	84.32%	84.27%	84.17%	83.88%	83.25%	
Positioning time	3.28 s	2.81 s	2.46 s	2.19 s	1.99 s	

### Influence of the size selection of the positioning area based on the nearest neighbor method on the positioning performance

**FIGURE 4:**
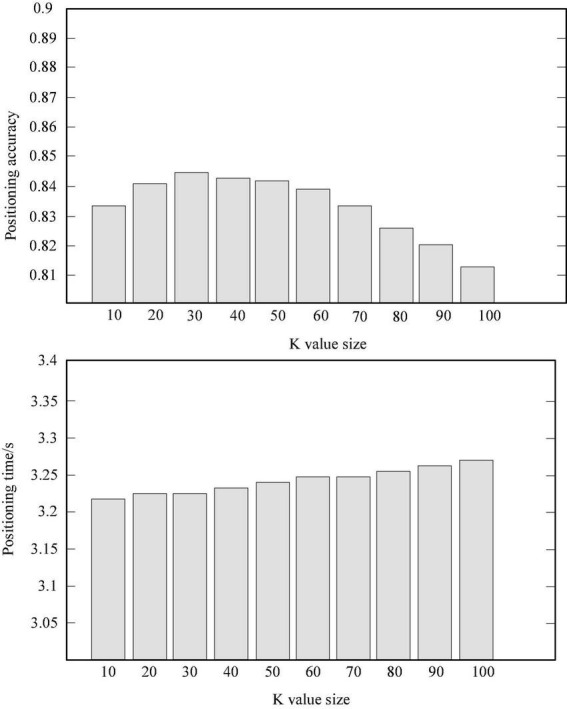
The relationship between the K value of the KNN algorithm and the positioning speed and accuracy.

It trains the training dataset with the 20,341,920 driving test data described in Figure 2 and finds a dataset containing 1,521,032 driving test data. The closest is set to 30, and the side length of the combined training data region is set to 10 m. The simulation results are shown in Table 2 and Figure 4. The positioning accuracy percentage in the table is the percentage of the specified positioning error within 100 m (Li, 2019).

**TABLE 2:** The simulation results of the size of the positioning region based on the k-nearest neighbor method.

Location area side length	100 m	200 m	300 m	400 m	500 m
Positioning accuracy	80.92%	83.36%	84.32%	84.97%	85.20%
Positioning time	0.93*s*	1.82*s*	3.28*s*	4.92*s*	6.40*s*
Location result	12.215	5.625	9.652	11.325	15.621

**FIGURE 5:**
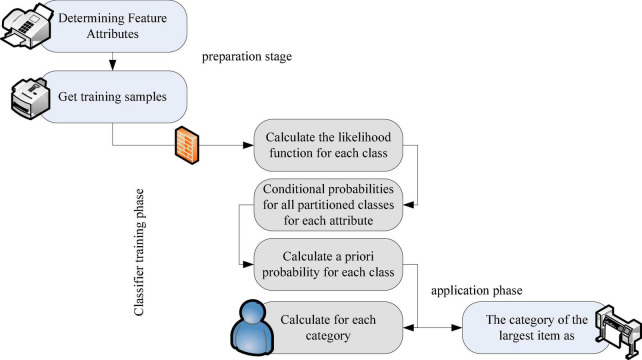
Bayesian classification diagram.

The angle of the problem in Bayesian analysis is the posterior distribution of the parameters, that is, the posterior distribution is obtained. If the original statistical samples and models are ignored and no longer considered, it will have little impact on future statistics, and make full use of the prior knowledge of sample information and parameters (Luo et al., 2021). Hypothesis testing is a statistical inference method used to judge whether the differences between samples and samples and between samples and the population are caused by sampling error or essential differences. Bayesian estimators quantify the outcome of hypothesis testing or estimation problems rather than simple judgments. With the exception of confidence intervals, instantaneous estimates, and large statistical tests, they no longer fall into the category of Bayesian inference. The steps of Bayesian classification application are summarized as the application of Bayesian network distribution specific classification must be completed in three stages (Jiang and Jiang, 2021): The first step: the preparation stage, manual operation, specifying feature attributes, appropriate division, and forming a set of training samples. The second stage: the training part, which automates the computations, computes conditional frequency and probability estimates, and records the results. The third stage: the application stage, the procedure is completed mechanically and the distributed items are distributed. The Bayesian classification process is shown in Figure 5.

**FIGURE 6:**
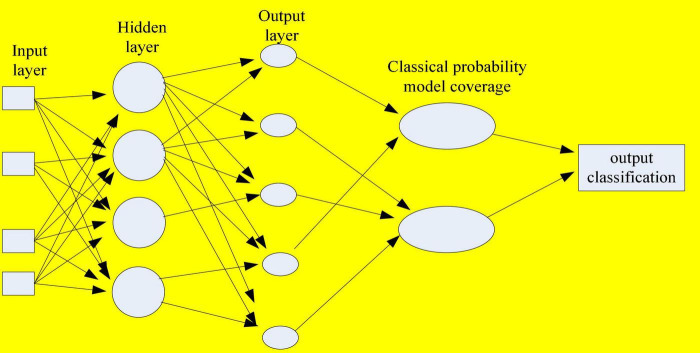
Bayesian probabilistic model classification learning internal model.

The following is designed from two aspects of external and internal models, in which the external model is the functional model, and the internal model is the mechanism structure design. The model is a covering algorithm based on classical probability and Bayesian probability, and belongs to a two-layer sub-module hybrid neural network structure (Wu et al., 2022). It consists of a network of first and second modules, where the first module implements the first classification of the dataset. There are two types of classification results. The data sets with overlapping classifications are further processed to add knowledge of the posterior probability. It uses Bayesian neural network for secondary classification to achieve optimal classification results, and finally determines the classification to which it belongs. The specific structure is shown in Figure 6. The steps of the coverage algorithm based on Bayesian theory are based on the idea of coverage algorithm based on Bayesian theory. According to the results of the first-level neural network processing, the overlapping coverage of the learning samples in the coverage is used. The prior probability that the heterogeneous points belong to a certain coverage classification calculated with Bayesian formula to get its posterior probability to assist judgment. Finally, the final category of the sample is determined, and the classification is output to complete all the classification tasks.

### Data collection and preprocessing

We define three attribute features: location matching index, activity index and influence index. These features are obtained by appropriate calculation and transformation of user information in the user information list, and the location matching index is used to measure the similarity of the user’s location. The activity index is used to measure the total activity of two users in Sina Weibo, and the influence index is used to measure the influence of the two users as the object of attention.


(1) $$L_{p}=n_{1}\cdot \theta_{1}$$

(2) $$L_{r}=n_{2}\cdot \theta_{2}$$

(3) $$F_{i\,m\,c}\,\left(u,v\right)=L_{p}\,\left(v\right)\cdot L_{r}\,\left(u\right)$$

Among them, C represents the name of the city where the user is located, and D represents the name of the region where the user is located. Obviously, the location matching index is an undirected feature.


(4) F⁢l⁢m⁢c⁢(u,v)=0,i⁢f,C⁢(u)≠C⁢(v),D⁢(u)≠D⁢(v)

(5) F⁢l⁢m⁢c⁢(u,v)=1,i⁢f,C⁢(u)=C⁢(v),D⁢(u)≠D⁢(v)

(6) F⁢l⁢m⁢c⁢(u,v)=2,i⁢f,C⁢(u)=C⁢(v),D⁢(u)≠D⁢(v)

Intuitively, the greater the total activity of the two users, the greater the scope of the two users, and the more likely the two users will follow each other. For any directed user pair (u, v), then define the active index of two users as:


(7) $$F_{a\,c}\,\left(u,v\right)=\frac{2\cdot \left(n_{1}+m_{1}\right)+\left(n_{2}+m_{2}\right)}{100}$$

(8) $$F\,i\,c\,\left(u,v\right)=\frac{\sum_{i=1}^{100}\left(n_{i}+m_{i}\right)}{100}$$

Let the set of all neighbor nodes of node v∈V be:


(9) Γ⁢(v)={u|⟨v,u⟩∈E⁢o⁢r⁢⟨u,v⟩∈E}

(10) $$\Gamma_{o\,u\,t}\,\left(v\right)=\left\{u|\left\langle u,v\right\rangle \in E\right\}$$

(11) $$\Gamma_{i\,n}\,\left(v\right)=\left\{u|\left\langle u,v\right\rangle \in E\right\}$$

(12) $$d_{i\,n}\,\left(v\right)=\left|\Gamma_{i\,n}\,\left(v\right)\right|$$

Assuming that the connections between users in a social network are randomly generated, the probability of two users being connected within a specific time should be proportional to the product of the number of neighbor nodes of the two users. The preference connection is formally proposed based on the above conclusion, and its definition is as follows:


(13) S⁢(u,v)={⟨x,y⟩∈E|x,y∈Γ⁢(u)⁢⋃Γ⁢(v)}

(14) $$F_{t\,f}\,\left(u,v\right)=\left|\Gamma \,\left(u\right)\,⋃\Gamma \,\left(v\right)\right|$$

(15) $$F_{j\,c}\,\left(u,v\right)=\frac{\left|\Gamma \,\left(u\right)\,⋂\Gamma \,\left(v\right)\right|}{\left|\Gamma \,\left(u\right)\,⋃\Gamma \,\left(v\right)\right|}$$

(16) $$F_{p\,a}\,\left(u,v\right)=\left|\Gamma \,\left(u\right)\right|\cdot \left|\Gamma \,\left(v\right)\right|$$

Based on the assumption that the more links between the neighbor nodes of two users in a social network, the greater the possibility of two users generating links, the friend metric is defined as follows:


(17) $$F_{f\,m}\,\left(u,v\right)=\sum_{x\in \Gamma \,\left(u\right)}\sum_{y\in \Gamma \,\left(v\right)}\delta \,\left(x,y\right)$$

(18) δ⁢(x,y)=1,i⁢f,x=y,o⁢r⁢⟨x,y⟩∈E⁢o⁢r⁢⟨y,x⟩∈E

(19) δ⁢(x,y)=1,i⁢f,x=y,o⁢r⁢⟨y,x⟩

(20) δ⁢(x,y)=0⁢o⁢t⁢h⁢e⁢r⁢w⁢i⁢s⁢e

## Theoretical and hypothesis on psychological expectation value of cultural tourism tourists

**FIGURE 7:**
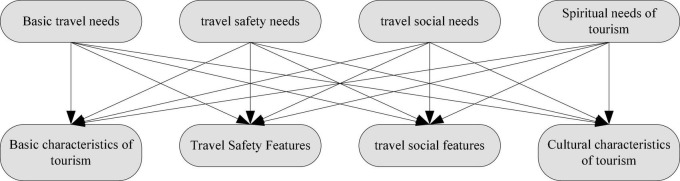
Travel destination product feature configuration.

The evaluation of tourists’ psychological expected value of a tourist destination is the subjective expected cognition of tourists on the tourism value of a tourist destination, which is different from the objective value of a tourist destination. When tourists choose a tourist destination, it mainly depends on the psychological expected value. Quality Function Configuration (OFD) is a product development method that reflects the voice of customers. It is an effective tool for the product development team to configure product functional characteristics, production technology and quality control technology according to customer needs. In the evaluation of destination tourists’ psychological expectation, tourists evaluate the psychological expectation value of destination tourism according to the known characteristics of destination tourism products. Therefore, it is necessary to establish the quality house of destination tourists’ psychological expectation evaluation, and clarify the relationship between tourists’ needs and the characteristics of destination tourism products. According to the known characteristics of destination tourism products, the psychological expected value of tourists to the destination is obtained, and a reasonable choice of tourism destination is made. Different tourists or groups of tourists have different tourism needs and the importance of tourism needs. Before traveling, the needs of tourists must be analyzed hierarchically, which is represented by Y-Y. Basic travel needs include travel, travel, accommodation, food and shopping needs. Tourism safety needs include the needs for the safety of the destination, such as nature, transportation, and society, tourism social needs include the needs for friendship, love, and affiliation, and tourism spiritual needs include entertainment, culture, and other needs. The next step is to determine the weight of visitor demand, and select the relevant tourist destination product characteristics according to the needs of tourists. The characteristics of tourism destination products include basic tourism characteristics, tourism safety characteristics, tourism social characteristics and tourism cultural characteristics. These characteristics are the characteristics of the comprehensive performance of various tourism products in the tourism destination. The tourism products include sightseeing tourism products, holiday tourism products, special tourism products, ecotourism products and tourism safety products. The quality house of destination tourists’ psychological expectation evaluation only needs to select the relevant tourism destination product characteristics according to the needs of tourists, which is represented by X-X. One tourist demand may correspond to multiple product features, and multiple tourism demands may correspond to one product feature, as shown in Figure 7. Tourism needs can be divided into four categories, physical needs, human needs, divine needs and social needs.

When the control variable is added according to the hypothesis, that is, the emotional information that tourists see, there are two situations: one is that the perceived positive information is greater than the negative information, and the other is that the negative information is seen more than the positive information. SPSS software was used for partial correlation analysis, and the influence of comprehensive emotional bias information was used for comparative analysis. As shown in Table 3, emotional bias information may have a controlling effect on variables and have a specific effect on the correlation of each variable. Better validation and analysis of the control variables using AMOS software revealed that when the overall sentiment bias in the information differs, there are different consequences in shaping visitor expectations, visitor satisfaction and attitudes. Emotional bias information appears to be a latent variable affecting tourist satisfaction and behavior, and has special significance as a control variable for the analysis.

**TABLE 3:** Partial correlation analysis of sentiment synthesis bias.

Control variable			Information	Expected mean	Satisfaction mean
None	Information	Correlation	1.000	0.437	0.484
	Expected mean	Correlation	0.437	1.000	0.702
	Satisfaction mean	Correlation	0.484	0.702	1.000
	Comprehensive attitude	Correlation	0.281	0.566	0.553
Synthetic bias of information emotions	Comprehensive bias	Correlation	0.011	0.248	0.193
	Information	Correlation	1.000	0.449	0.491
	Expected mean	Correlation	0.449	1.000	0.688
	Satisfaction mean	Correlation	0.491	0.688	1.000
	Comprehensive attitude	Correlation	0.285	0.543	0.534

After the group discussion, the purpose of the group tour is to enjoy leisure and entertainment tourism while viewing the culture of the houses, and the purpose is to release the work pressure. The tourist demand of tourists is convenient transportation, beautiful waterscape, shady trees, hygienic catering, no crowding, excellent residential culture and complete rest facilities, as indicated by TR-TR. Group tourists use AHP to determine the weight W of tourists’ demand, as shown in the left-hand side of Table 4. It selected and evaluated tourist destination product characteristics and characteristic values according to tourist needs, expressed by TC-TC. Tourism experts evaluate the product characteristic values of 5 tourist attractions between 0 and 1. 0–1 means from worst to best as shown in the lower right of Table 4 (Woodward, 2006).

**TABLE 4:** The quality of psychological expectation assessment of tourists in the class destination.

		Tourism destination product characteristics							
	TC1	1	0.33	0	0	0	0	0	0
	TC2	0.33	1	0	0.33	0	0.33	0	0
	TC3	0	0	1	0.33	0	0	0	0.11
	TC4	0	0.33	0.33	1	0	0	0	0
	TC5	0	0	0	0	1	0.11	0	0
	TC6	0	0.33	0	0	0.11	1	0	0.33
	TC7	0	0	0	0	0	0	1	0.11
	TC8	0	0	0.11	0	0	0.33	0.11	1

**Tourist demand**		**TC1**	**TC2**	**TC3**	**TC4**	**TC5**	**TC6**	**TC7**	**TC8**

TR1	0.15	1	1	0	0	0	0	0	0
TR2	0.13	0	0	1	0.33	0	0	0	0
TR3	0.25	0	0	0.33	1	0	0	0	0.33
TR4	0.20	0	0	0	0	1	0.33	0	0.11
TR5	0.13	0	0.33	0	0	0.33	1	0	0.11
TR6	0.25	0	0	0	0.11	0	0	1	0.11
TR7	0.07	0	0	0.11	0.33	0	0.11	0	0
	X1	0.45	0.80	0	0	1	0	0.87	0.30
	X2	0.90	1	0.40	0.30	0.40	0.25	0.80	0.42
	X3	0.80	0.85	0.75	0.80	0.50	0.85	0.30	0
	X4	0	0.50	0.60	0.60	0	1	1	0.34
	X5	1	0.80	1	1	0.20	0.90	0	1

**FIGURE 8:**
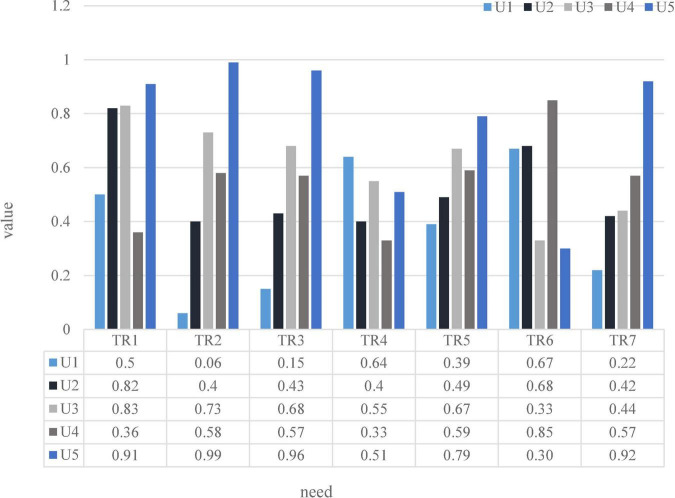
Tourist demand value of tourist attractions.

Experts evaluate the relationship between tourist demand and product characteristics of tourist destinations, and the autocorrelation relationship between product characteristics. The experts in the House of Integrated Tradeoffs assessed the relationship between tourist demand and product characteristics of tourist destinations, as well as the autocorrelation relationship between product characteristics. The tourist demand value of each tourist attraction calculated according to the formula is shown in Figure 8:If the price of the scenic spot is high, the expectations for the tourist attraction will also increase accordingly, and any unexpected circumstances in the itinerary may affect the magnitude of the experience; if the price of the scenic spot is low, the scenic spot may not be completed, and the profit will be reduced. Therefore, it is particularly important for scenic spots to establish a reasonable price control system and standardize the price standards of various charging items in scenic spots. Flexible pricing strategies can be implemented according to the life cycle and market characteristics of tourist attractions, attractive prices, such as working group tickets, family tickets, etc., or the introduction of special holiday tickets for tourists; it is also possible to bundle the charges for tickets, sightseeing services and other facilities through price transfer and linkage pricing strategies, shift the focus, and expand income, such as tickets, food and accommodation, and form preferential travel packages to choose from. Lucky tourists are picked up here every day, and tickets or restaurant vouchers of different values are provided for sightseeing purchases. All transportation, meals and lodging are attached to the ticket. As a coupon for affiliates, in addition, there are different, flexible pricing strategies, such as discounts on tickets booked by phone or online. According to the analysis of the previous survey results, tourists stay in the scenic spot for about an hour, and tourists generally use tickets, and some use and consume things. According to the specific situation, the scenic spot can first strengthen the catering and accommodation services in the park to guide tourists to use it; the second is to increase the variety of tourism products, improve the quality of goods, highlight local characteristics, and stimulate tourists’ desire to buy. To develop high value-added tourism products related to theme parks, when conditions are ripe, it can enter the original development of film and television animation. Through the above steps, the use of tourist tickets in the scenic spot can be transformed into the comprehensive use of food, housing, transportation, shopping and entertainment, which is convenient to increase the number of tourists and per capita tourism consumption.

## Discussion

The tourism information that tourists see is all the changes in the entire tourism project chain, which is also the beginning of all tourism projects. Through a lot of in-depth research, tourism data and traveler habits are well integrated into the entire tourism industry, and it is estimated that tourism data will have an impact on all stages of the tourism industry. Data analysis shows that tourists’ perception of tourist information is closely related to tourists’ expectations of their location, that is, tourist data directly affects the composition of tourists’ expectations. It is worth noting that tourist attractions have increased the promotion of tourist information, so tourists are optimistic about the prospects of tourist attractions. This study shows that travel information is significantly correlated with passenger satisfaction and travel identification. Age affects visitor data, however, travel data does not have the same impact on visitor expectations and satisfaction. Therefore, to understand the prospects of tourist attractions, the image of tourism will depend on the actual local situation. Taken together, the impact of travel data on travel habits is not significant, but indirect factors such as expectations and satisfaction will have an impact. Because the attitude of tourists in the tourism industry changes due to factors such as expectations and satisfaction, the role of tourism information gradually declines. For some tourist destinations that are economically infeasible and do not have advertisements, you can try to improve the quality of local tourism services, so that customers can have a good time, experience, and leave a deep impression, create a good image, and use the behavior and feelings of tourists to communicate word of mouth. Finally, travel information is a double-edged sword, good or bad travel information can be transmitted through instant messaging. Tourists are more sensitive to negative information than to good information, and tourist information is evenly distributed, forming a stereotype in tourists’ minds that can reduce the appearance of bad news. Tourism information is very convenient and has the function of managing and improving the tourism market.

At present, comprehensive information about the city’s service industry is also released soon, which makes travel information easier and more convenient. Travelers need travel information to develop routes, view digital maps, test real-time searches, enjoy online bookings and share travel experiences. Travel information is everywhere in travel planning. Popularity, self-organization, and personal travel have become modern hobbies. Self-driving, backpacking, independent travel, business travel, and more are commonplace. This is a huge market, and there are many things that require smart and efficient customized services and data.

Therefore, tourists should evaluate tourism information services and tourist areas, and increase the importance of releasing tourist information to tourist destinations to create value for shopping and managing tourist destinations. Tourist and sightseeing guides, marketing and tourism promotion should establish and develop customer-centric information systems that provide timely and effective information on sightseeing and potential sightseeing tours, also contributing to increased visitor expectations, visitor satisfaction and overall ethical development. In today’s convenient information network, all kinds of information can be easily disseminated. Tourism enterprises need to reform and innovate to adapt to the era of big data and information.

## Conclusion

From the perspective of tourists, this paper has important theoretical and practical significance to study the psychological expected value of tourists to tourist destinations. It selects the correct travel plan to meet the individual needs of tourists, improve travel satisfaction, and achieve effective allocation of resources. The quality function allocation method can weigh the relationship between tourist demand and product characteristics of tourism destinations, and the autocorrelation relationship between the characteristics of tourism destination products. The psychological expectation evaluation model of tourists in tourist destinations can effectively calculate the perceived value of the psychological expectation evaluation of tourist destinations according to the tourist needs of tourists, combined with the product characteristics of tourist destinations and the knowledge and experience of tourism experts, and help tourists choose reasonable and satisfactory travel plans. Through ML and DM techniques, we study the evaluation of tourists’ psychological expectations. For the positioning of mobile terminals outside the Internet, this paper imitated two traditional positioning methods based on ML. The deployment method based on ML, through the sequential training data preprocessing and comprehensive development of traditional ML algorithms, the deployment accuracy is significantly improved, and the deployment time complexity is reduced. Investigate tourism information from the perspective of tourists. According to the information dissemination mechanism, the role of tourism information is also greatly influenced by information disseminators and information publishers. Therefore, follow-up research should add factors and variables of tourism information, integrate the comprehensive function of information into tourism activities, and make assumptions and models more perfect. In addition, the research on tourist behavior in this paper can further analyze the impact of various behavioral variables and emotional aspects on potential tourists. Finally, sequence studies can add hidden variables, such as sources of information channels, as control variables to better assess their impact on tourism behavior.

## Data availability statement

The original contributions presented in this study are included in the article/Supplementary material, further inquiries can be directed to the corresponding author.

## Author contributions

C-HP collected and analyzed the data and drafted the manuscript. SX and JJ designed the research protocol and contributed to the literature review. YS reviewed and revised the manuscript. All authors listed have made a substantial, direct, and intellectual contribution to the work, and approved it for publication.
